# In the September 2012 issue

**DOI:** 10.1590/S1980-57642012DN06030001

**Published:** 2012

**Authors:** Sonia M.D. Brucki

**Affiliations:** Associate Editor

In this month's issue we have a number especially devoted to vascular cognitive impairment. This concept encompasses from brain at risk to vascular dementia. A huge volume of knowledge is produced worldwide on this issue. We are publishing fours reviews, seven original papers and two case reports.

**Grinberg** presented a discussion on current nomenclature for cerebrovascular lesions in pathological fields. There is an overlapping of nomenclature among different studies concerning vascular pathological findings in the brain.

**Dutra** produced a review on carotid stenosis and cognitive impairment. There are many concerns about grade of stenosis and cognition, side of stenosis, and the optimal therapy (stenting or endarterectomy).

**Damasceno** in this review discussed the relationship between cortical microinfarcts and cognition. This manuscript outlines possible mechanisms by which microinfarcts cause cognitive impairment, such as hypoperfusion, hypoxia, oxidative stress, and inflammation with possible disruption of cognitive networks.

**Vale et al.** reviewed vascular Parkinsonism and cognitive impairment. They present studies performed in Brazil, with a range of vascular parkinsonism from 2.3 to 15.1% in elderly cohorts. Symptoms and practical issues on vascular parkinsonism are addressed.

**Sudo et al.** conducted a review regarding vascular mild cognitive impairment. The main syndrome seen in these patients is dysexecutive syndrome associated with strokes and periventricular and white matter hyperintensities on neuroimaging.

**Terroni et al.** performed a systematic review about post-stroke depression and cognition. The occurrence of this condition is frequent after stroke, varying from 22 to 31%. Vascular depression is associated with increased cognitive impairment, reported in 35 to 87% of depressed patients.

**Alves et al.** reviewed the role of diffusion tensor imaging as a biomarker for monitoring vascular disease progression and integrity of white matter tracts, and correlation with cognitive impairment.

**Brucki et al.** investigated the prevalence of vascular cognitive impairment in a sample of post-stroke patients during a follow-up of 12 months. Among 172 stroke patients, 12.2% had a diagnosis of dementia and 4.6% of cognitive impairment no dementia.

**Matioli & Caramelli** analyzed possible different profiles between subcortical vascular dementia and Alzheimer's disease patients using the NEUROPSI battery. Alzheimer's disease patients performed worse on verbal fluency and memory recall items of the NEUROPSI.

**Tiel et al.** described 50 patients with extensive white matter hyperintensities. Patients with mild-moderate to severe hippocampal atrophy presented worse cognitive and functional performance in relation to the group with no or questionable hippocampal atrophy.

**Oliveira-Filho et al.** provided a description about the rationale and methodology of an important study with an estimated sample size of 500 Chagas disease patients (CLINICS) and preliminary results of the first 90 evaluated patients.

**Da Silva et al.** reported a case of Cerebral Autosomal Dominant Arteriopathy (CADASIL) and the characteristic neuropsychological profile and neuroimaging related to this early onset vascular dementia.

**Siqueira-Neto et al.** reported a case of vascular cognitive impairment associated with *pemphigus vulgaris* and comment on differential diagnosis for cognitive impairment in patients with auto-immune conditions.

**Sonia M.D. Brucki**

Associate Editor

